# An investigation of Li_2_TiO_3_–coke composite anode material for Li-ion batteries†

**DOI:** 10.1039/c9ra02611h

**Published:** 2019-06-05

**Authors:** Youlin Liu, Wensheng Li, Xiaoping Zhou

**Affiliations:** Department of Chemical Engineering, Hunan University Changsha Hunan 410082 PR China hgx2002@hnu.edu.cn

## Abstract

Anode material Li_2_TiO_3_–coke was prepared and tested for lithium-ion batteries. The as-prepared material exhibits excellent cycling stability and outstanding rate performance. Charge/discharge capacities of 266 mA h g^−1^ at 0.100 A g^−1^ and 200 mA h g^−1^ at 1.000 A g^−1^ are reached for Li_2_TiO_3_–coke. A cycling life-time test shows that Li_2_TiO_3_–coke gives a specific capacity of 264 mA h g^−1^ at 0.300 A g^−1^ and a capacity retention of 92% after 1000 cycles of charge/discharge.

## Abstract 2

Anode material Li_2_TiO_3_–coke was prepared and tested for lithium-ion batteries. The as-prepared material exhibits excellent cycling stability and outstanding rate performance.

## Graphical abstract

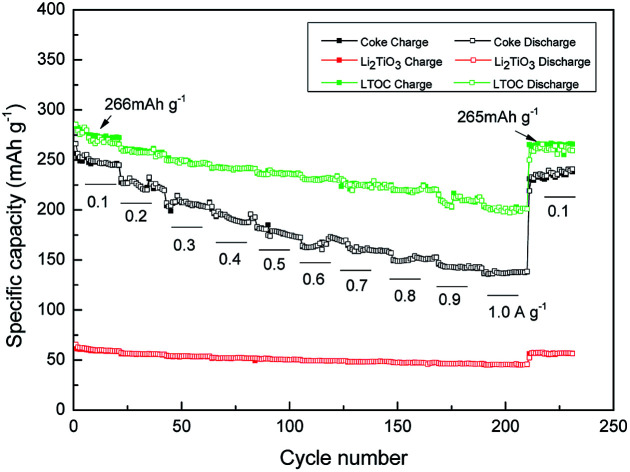

## Introduction

Except for high energy density, long cycling life-time, and high safety, the application of rechargeable batteries in electric vehicles also requires that the batteries have high power density. In the manufacture of high-power density batteries, high rate capable cathode and anode materials are required.^1,2^ Generally, the cathode materials, such as NCM and LiFePO_4_, are high rate capable. However, an anode material with high rate capability and high energy density is still not available. Presently, graphite is widely employed as an anode material in lithium ion batteries.^3,4^ However, graphite is not high rate capable.^5^ The spinel lithium titanate (Li_4_Ti_5_O_12_) is a high rate capable anode material, which has an almost zero-strain.^6^ The reduced volume change in lithium ion intercalation/deintercalation guarantees its high reversibility^7,8^ and long cycling life-time. The high lithium insertion potential (about 1.55 V *vs.* Li^+^/Li) could effectively avoid lithium plating over the anode in the charge process even at low temperature or high current density.^9,10^ Hence, regarding safety issues, Li_4_Ti_5_O_12_ represents a much better option than graphite. However, except the advantages of the spinel Li_4_Ti_5_O_12_, the high lithium ion insertion potential and the relatively low theoretical capacity (175 mA h g^−1^) of Li_4_Ti_5_O_12_ lead to a lower cell energy density, which limits the applications of Li_4_Ti_5_O_12_ in areas that need the batteries having higher energy density. Hence, searching for high performance anode material that has high specific capacity, high rate capability, long cycling life-time, and high safety is still the goal of researchers in battery investigation.

Monoclinic Li_2_TiO_3_ has a layer structure and a three-dimensional lithium ion diffusion network.^11^ In recent years, Li_2_TiO_3_ is often added into cathode materials, such as LiNi_1−*x*−*y*_Co_*x*_Mn_*y*_O_2_ (*x* > 0, *y* > 0, *x* + *y* < 1) and LiCoO_2_, to improve their rate capability and stability.^12,13^ Although, Li_2_TiO_3_ is a good lithium ion conductor, it is almost an electronic insulator. In order to making use of Li_2_TiO_3_ as an anode material, one must improve the electronic conductivity of Li_2_TiO_3_. Investigators usually add carbon materials^14–18^ into metal oxide electrode materials or directly prepare the metal oxide electrode materials in metal foam^19,20^ to improve their electronic conductivity. Since the electronic conductivity of Li_2_TiO_3_ is very poor, we choose to support relatively less amount of Li_2_TiO_3_ over relatively larger amount of coke to reach good electronic conductivity. Because petroleum coke has medium electronic conductivity and pores that lithium ions could easily diffuse in and out. The coke itself is a potential high rate anode material. In the present work, by making use of the good lithium ion conductivity of Li_2_TiO_3_ and the electronic conductivity of coke, we intend to use lithium hydroxide, TiO_2_, and petroleum coke to prepare a practically useful high rate anode material Li_2_TiO_3_–coke (LTOC) for lithium ion batteries.

## Experimental

### The synthetic procedure of Li_2_TiO_3_

Li_2_TiO_3_ was prepared by the following method. TiO_2_ (5.6724 g), LiOH·H_2_O (5.9602 g), and H_2_O (50.0 ml) were mixed and milled at 180 rpm for 6 h to obtain a slurry. The slurry was transferred into a beaker. The milling container and beads were washed three times with DI water (10 ml for each time) and the washing out mixture was combined with the previous slurry to obtain a diluted slurry. Adding DI water to the diluted slurry to a total volume of 100 ml for spray dry. After spray dry, a powder was obtained, and then the powder was calcined at 850 °C for 4 h in air to obtain Li_2_TiO_3_.

### The pretreatment of oil coke

The oil coke was placed into a tube furnace and heated from room temperature to 1200 °C at a heating rate of 5 °C min^−1^ in argon, then heated at 1200 °C for 6 h, and then cooled down to room temperature in argon to obtain the preheated coke, which was used as anode material and precursor for Li_2_TiO_3_–coke (LTOC) preparation.

### The synthetic procedure of LTOC

The LTOC was prepared through the following method. The preheated coke (6.5000 g), TiO_2_ (1.7067 g), LiOH·H_2_O (1.7933 g), surfactant (([*n*-C_16_H_31_(CH_3_)_3_N]CO_3_CH_3_) writing as QA, 0.9457 g), and distilled water (50.0 ml) were added into a ball mill container and milled at 180 rpm for 6 h to obtain a slurry. The slurry was transferred into a beaker. The mill container and beads were washed three times with DI water (10 ml for each time) and the washing out mixture was combined with the previous slurry to obtain a diluted slurry. Adding DI water to the diluted slurry to a total volume of 100 ml for spray dry. After spray dry, a powder was obtained, and then the powder was calcined at 850 °C for 4 h in argon to obtain LTOC.

### Cell preparation

The as-prepared anode material, conductive carbon black, carbon nanotubes (dispersed in *N*-methyl-2-pyrrolidone), polyvinylidene fluoride (PVDF) binder (dissolved in *N*-methyl-2-pyrrolidone) were mixed according to the mass ratio of 85 : 10 : 1.5 : 3.5 to prepare a paste. The paste was coated on a copper foil (the thickness of the anode coating layer is 30 μm) with an applicator (frame-type), and then dried in a vacuum oven at 120 °C for 2 h to obtain the anode plate. The dried anode plate was cut into cycles with a diameter of 14 mm. The anode cycles were farther dried in a tubular furnace (filled with argon) at 140 °C for 5 hours, and then transferred into an argon filled glove box (Mikrouna) in argon atmospheres. The anode cycles together with the separator (PP), the lithium metal cycles (as the counter electrode), and the electrolyte solution (1.0 M of LiPF_6_ in EC, PC, DMC, and EMC, the volume ratio of EC, PC, DMC, to EMC is 1 : 1 : 1 : 1) were used to assemble coin-type cells.

The crystal structure of the samples was analyzed by X-ray diffraction spectroscopy (Rigaku D/Max-rA, CuK radiation). The surface morphology and microstructure of the samples were characterized by SEM (FE-SEM S-4800, Hitachi) and TEM (Titan G2 60-300, FEI). Laser Raman measurements were conducted on a laser Raman spectroscopy meter with an excitation wavelength of 532 nm (*via* RM10000, Renishaw). The X-ray Photoelectron Spectroscopy (XPS) measurements of samples were conducted on a XPS spectroscopy meter (ESCALAB 250XI, Thermo Fisher). The specific surface areas of the materials were measured by BET method (Nova 2200e, Quantachrome).

The discharge/charge tests of the cells were carried out on a NEWARE CT-3008-5 V-10 mA system. The voltage window is 0.01–2.0 V. In the rate performance test, the cells were tested at current densities 0.033, 0.100, 0.200, 0.300, 0.400, 0.500, 0.600, 0.700, 0.800, 0.900, 1.000, 2.000, 3.000, 4.000, 5.000, and 0.100 A g^−1^, respectively. In the cycling life-time test, the cells were charge/discharged at 0.300 A g^−1^. The cycling voltammetry curves were recorded on an electrochemical workstation (CHI 660E, CHENHUA) at a scan rate of 0.1 mV s^−1^ within 0.01–2.0 V. The EIS measurement of cells was also carried out on the electrochemical workstation (CHI 660E, CHENHUA). All tests were carried out at room temperature.

## Results and discussion

The Laser Raman characterization shows that the preheated oil coke has 2 bands (the D band at 1360 cm^−1^ and the G band at 1580 cm^−1^). The ratio of the D band intensity (*I*_D_) to the G band intensity (*I*_G_) is 0.91 (*I*_D_/*I*_G_ = 0.91), which indicates that the preheated oil coke is not graphitized (ESI, Section 1 Fig. s1†).^21–23^ The TEM images also show that the preheated coke is not graphitized (ESI, Section 1 Fig. s2 and s3†).

The specific surface areas of Li_2_TiO_3_, coke, and LTOC are 2.9, 13.1, and 10.1 m^2^ g^−1^, respectively. These numbers are close to the specific surface area of regular Li_4_Ti_5_O_12_.^24^

In LTOC, the percentage of Li_2_TiO_3_ is 35.6 wt% (ESI, Section 2†). The EDS analysis indicates that the LTOC contains Al, Si, and S impurities, but not contains nitrogen (ESI, Section 3†).

**Fig. 1:**
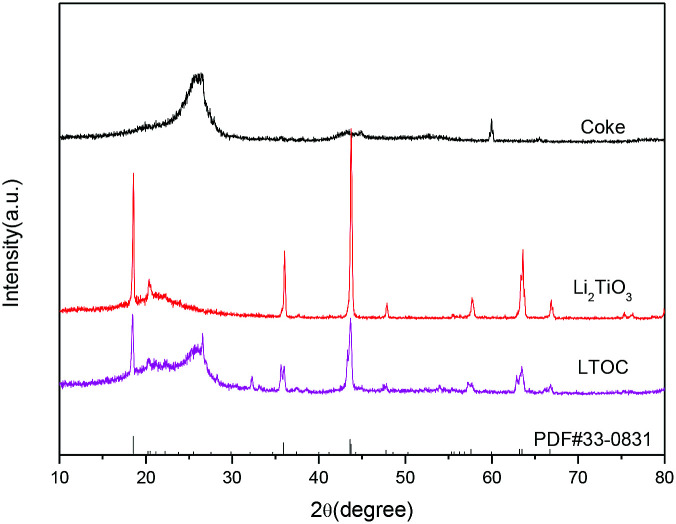
The XRD patterns of coke carbon, Li_2_TiO_3_, and LTOC.

The XRD analysis shows that the coke has only a broad peak between 20° and 30° (Fig. 1). The broad peak is assigned to the amorphous coke. The peaks of pristine Li_2_TiO_3_ match that of the standard monoclinic Li_2_TiO_3_ (PDF#33-0831) with lattice parameters *a* = 5.069 Å, *b* = 8.799 Å, and *c* = 9.759 Å. Hence, the pristine Li_2_TiO_3_ is monoclinic Li_2_TiO_3_. In LTOC, except from the broad peak between 20° and 30° (assigned to amorphous coke), there are peaks that exactly match that of monoclinic Li_2_TiO_3_. Hence, monoclinic Li_2_TiO_3_ is formed in LTOC.

**Fig. 2:**
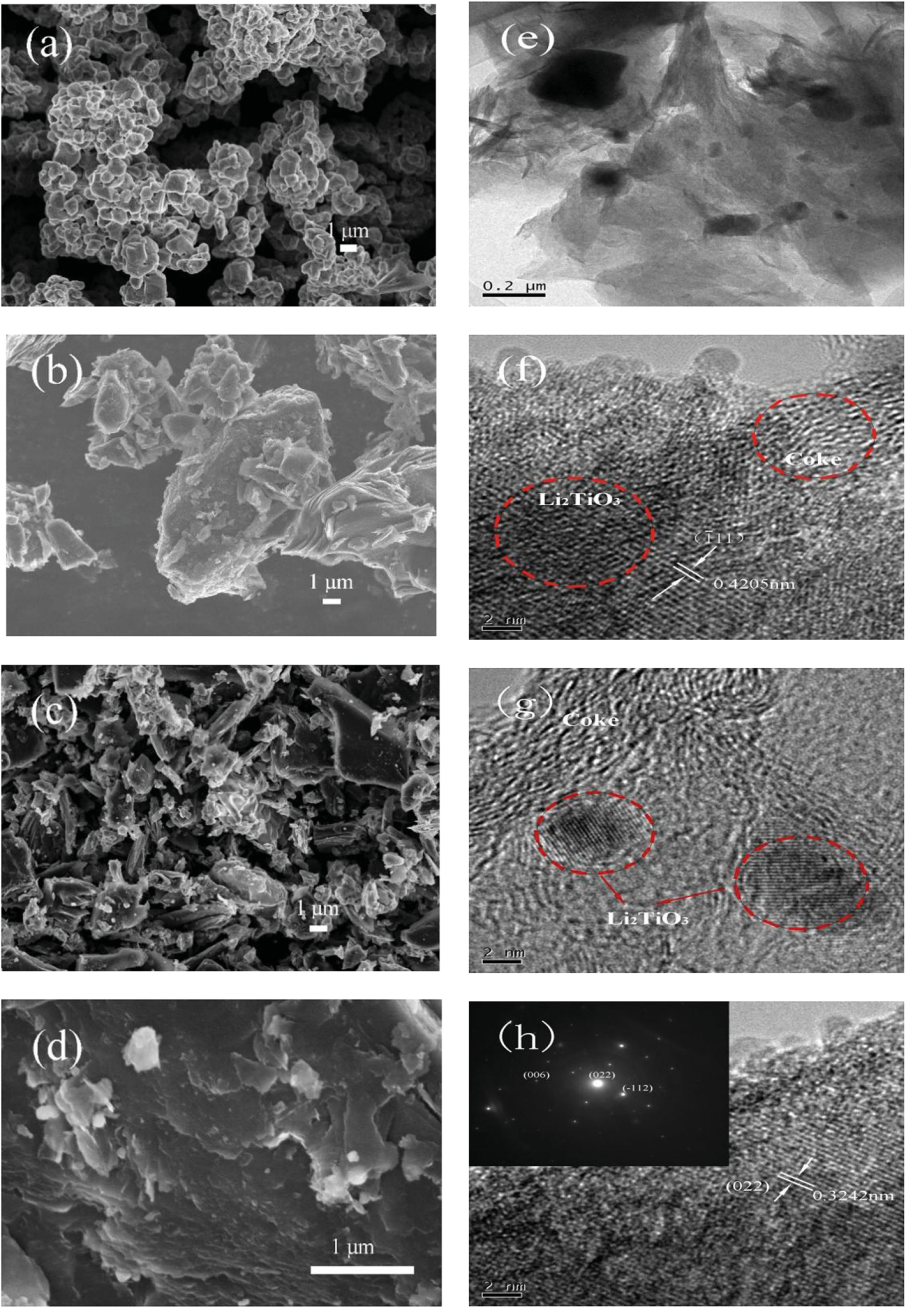
SEM images of Li_2_TiO_3_ (a), petroleum coke (b), and LTOC with different resolutions (c) and (d). TEM of LTOC (e), (f), (g), and (h).

The SEM images of Li_2_TiO_3_, coke, and LTOC are given in Fig. 2. The image of Li_2_TiO_3_ shows that the average size of Li_2_TiO_3_ crystals is about 1 μm. Petroleum coke has a particle size distribution from nanometers to tens of micrometers. In LTOC (Fig. 2c and d), the crystal size of Li_2_TiO_3_ is in nanometer level. The SEM images show that under the help of surfactant QA, the Li_2_TiO_3_ crystals are uniformly distributed on the coke particles. The results indicate that the presence of coke inhibits the growth of Li_2_TiO_3_ crystals. The attachment of Li_2_TiO_3_ nanocrystals on coke carbon could offer an excellent interface for electron exchange between Li_2_TiO_3_ and coke carbon. The EDS mapping of elements C, O, and Ti in LTOC (ESI Section 4 Fig. S4†) shows that the oxygen and titanium are uniformly distributed on coke carbon.

The high resolution TEM images (Fig. 2e–h) reveal that the Li_2_TiO_3_ crystals are formed on coke and the Li_2_TiO_3_ phase is tightly contacted with the carbon phase. The typical Li_2_TiO_3_ lattice spacings of 0.3242 and 0.4205 nm can be indexed to (022) and (111) planes, respectively (the inset of image (h) gives the selected area electron diffraction pattern of a Li_2_TiO_3_ crystal). The results are consistent with that observed in the SEM characterization.

**Fig. 3:**
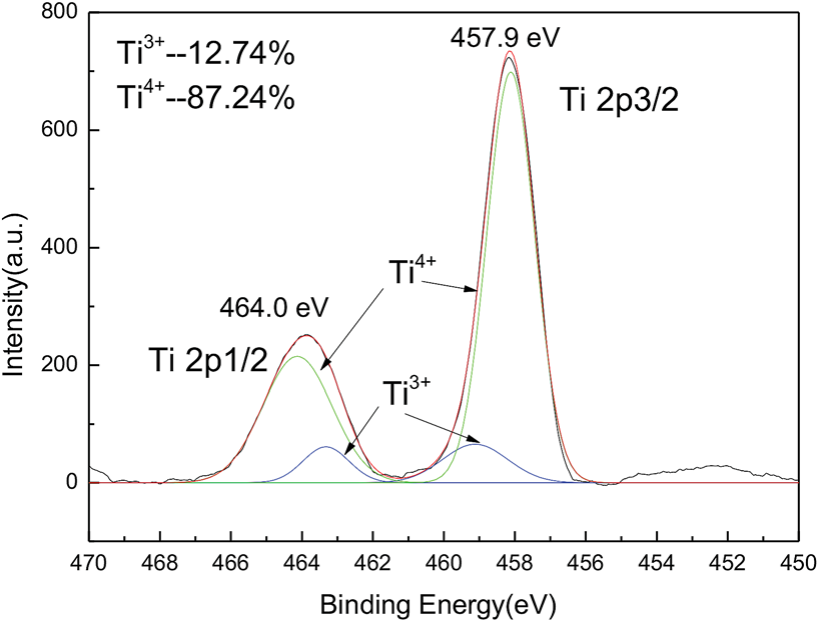
The XPS spectra of Ti 2p_1/2_ and 2P_3/2_.

The XPS measurement shows that there is about 12.7% of Ti^4+^ ions reduced to Ti^3+^ ions at high calcination temperature (850 °C). However, the major part of titanium ions is still T^4+^ ions (Fig. 3).

The cyclic voltammetry measurements are performed on fresh cells with metal lithium anodes. Fig. S5† (ESI Section 5†) shows the cyclic voltammograms (CVs) of Li_2_TiO_3_, coke, and LTOC in their first three cycles. In the reduction process, the CV curves of the three electrodes in their first cycle are significantly different from their subsequent curves, which could be caused from the SEI formation and irreversible structure change. Li_2_TiO_3_ has weak reduction/oxidation peaks at 1.52/1.63 V (Fig. S5a†), which are assigned to Li_2_TiO_3_.^25^ The major reduction/oxidation of Li_2_TiO_3_ appears between 0.01 and 1.00 V (*vs.* metal Li). The CV curves of coke (Fig. S5b†) have strong reduction/oxidation peaks between 0.01 and 1.30 V. The CV curves of LTOC is given in Fig. S5c.† The weak reduction/oxidation peaks of LTOC at 1.51/1.62 V are assigned to Li_2_TiO_3_. The major reduction/oxidation of LTOC is also observed between 0.01 and 1.30 V. The maximum reduction/oxidation currents on LTOC is bigger than that of coke and Li_2_TiO_3_, which indicates that more lithium ions could be inserted into LTOC or extracted from LTOC than coke and Li_2_TiO_3_.

From the CV data of coke, Li_2_TiO_3_, and LTOC, the lithium ion diffusion coefficient could be calculated through the Randles–Sevcik equation:

1 *I*_p_ = (2.69 × 10^5^)*n*^3/2^*AD*_Li^+^_^1/2^*C*_Li^+^_*V*^1/2^

The lithium ion diffusion coefficients (*D*_Li^+^_) of Li_2_TiO_3_ in the oxidation and reduction processes are 5.66 × 10^−8^ and 1.249 × 10^−8^ cm^2^ s^−1^, respectively (ESI Section 5†). The *D*_Li^+^_ of LTOC for the oxidation and reduction processes are 1.618 × 10^−8^ and 2.083 × 10^−9^ cm^2^ s^−1^, respectively. The *D*_Li^+^_ for the oxidation and reduction of coke are 1.54 × 10^−12^ and 4.078 × 10^−12^ cm^2^ s^−1^, respectively. The results show that the *D*_Li^+^_ of Li_2_TiO_3_ is bigger than that of LTOC and that of LTOC is bigger than that of coke. The addition of Li_2_TiO_3_ in coke favours the lithium ion diffusion in coke. The as synthesized LTOC also shows much higher *D*_Li^+^_ than the spinel Li_4_Ti_5_O_12_ (about 10^−9^ to 10^−13^ cm^2^ s^−1^).^26^

**Fig. 4:**
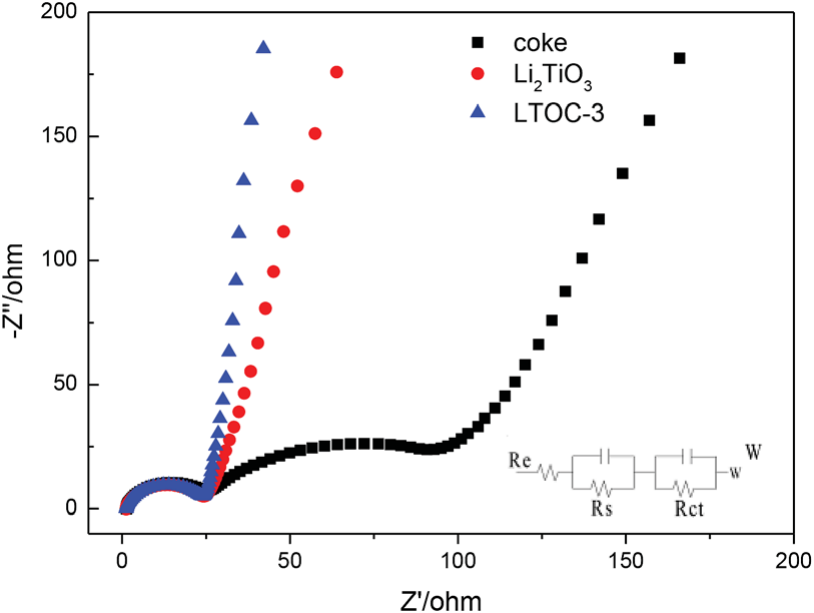
The EIS and the equivalent circuit for LTO, coke, and LTOC.

Fig. 4 shows the impedance spectra (EIS) of the materials. The Nyquist plots are fitted by using the equivalent circuit model. *R*_e_ stands for the resistance of the SEI membrane, *R*_s_ stands for the Li ion migration resistance, and *R*_ct_ stands for the charge transfer resistance.^27,28^ All the plots possess a depressed semicircle at high to intermediate frequency and an oblique line at low frequency. The results (ESI Section 6, Table S2†) show that Li_2_TiO_3_, coke, and LTOC have very close SEI resistance (*R*_e_) values (about 2.0 Ω). The *R*_s_ of LTOC (29.0 Ω) is smaller than that of coke (34.3 Ω), but close to that of Li_2_TiO_3_ (27.7 Ω). The results indicate that the Li^+^ diffusion rate in LTOC should be higher than in coke, but lower than in Li_2_TiO_3_. Among Li_2_TiO_3_, coke, and LTOC, the big differences in resistance are found in the charge transfer resistance. Coke has the smallest charge transfer resistance among Li_2_TiO_3_, coke, and LTOC (Table S2†*R*_ct_). The Li_2_TiO_3_ is almost an insulator to electrons. Although the addition of Li_2_TiO_3_ into coke reduces the electronic conductivity of coke (*R*_ct_ of LTOC), but improves the Li^+^ conductivity of the material. The *R*_e_ (2.01 Ω) and *R*_s_ (29.0 Ω) of LTOC are much smaller than the corresponding *R*_e_ (10.6 Ω) and *R*_s_ (79.6 Ω) of literature reported spinel Li_4_Ti_5_O_12_.^29^

**Fig. 5:**
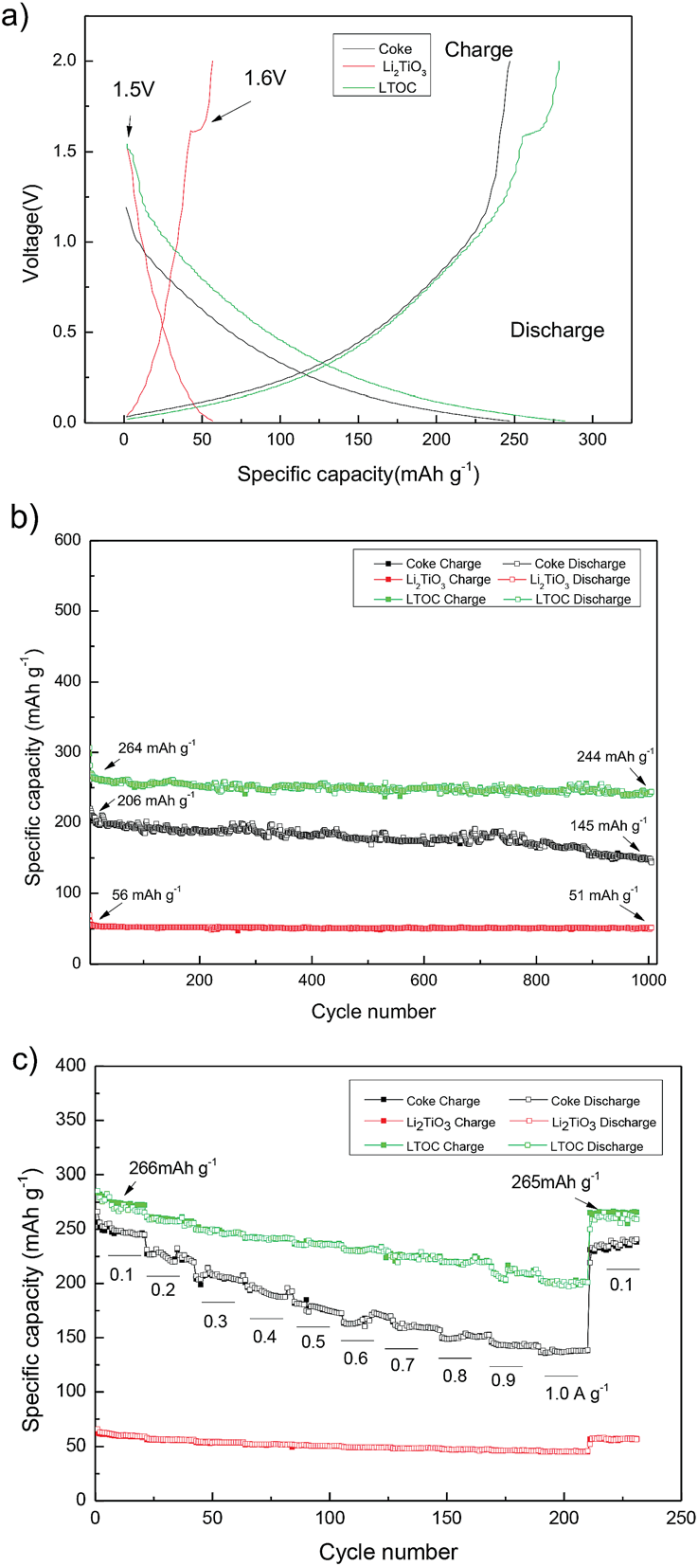
(a) The discharge/charge curves of the cells with coke, Li_2_TiO_3_, and LTOC as cathode and metal Li as anode at 0.100 A g^−1^; (b) discharge/charge cycling life-time test at 0.300 A g^−1^; (c) discharge/charge rate cycling test at different current densities.

Fig. 5a gives the discharge/charge curves of the cells with topic materials as cathode *vs.* lithium metal anode. The coke cell has smooth discharge/charge curves. The discharge/charge curves of LTOC and Li_2_TiO_3_ cells have a very narrow discharge voltage plateau (10–20 mA h g^−1^) at about 1.5 V and a narrow charge voltage plateau at about 1.6 V, which is contributed by Li_2_TiO_3_,^25^ while the rest parts of the curves are smooth. This type of smooth discharge/charge curves offers people options for selecting any suitable final charge voltage in full cells to avoid lithium plating on anodes. It could be found that, over LTOC, the most part of the discharge/charge occurs below 1.5 V. Hence, by comparing with the spinel Li_4_Ti_5_O_12_ (major discharge/charge voltage plateau at about 1.55 V), the batteries having LTOC anode should give much higher full cell voltage and energy density than that having a Li_4_Ti_5_O_12_ anode (ESI Section 7†).

Fig. 5b shows the cycling performance of cells with Li_2_TiO_3_, coke, and LTOC anodes at 0.300 A g^−1^. The Li_2_TiO_3_ retains 92.8% of it's initial specific capacity after 1000 discharge/charge cycles, indicating very good stability. However, the specific capacity of Li_2_TiO_3_ is low (56 mA h g^−1^). Coke has higher specific capacity (initially 206 mA h g^−1^) than Li_2_TiO_3_, but bad stability. Coke only retains 68.5% of it's initial specific capacity after 1000 discharge/charge cycles. The LTOC cell performances the best. The LTOC has the highest specific capacity of 264 mA h g^−1^. After 1000 of discharge/charge cycles, LTOC still retains 92.4% of it's initial specific capacity.

Fig. 5c shows the rate performances of LTOC, coke, and Li_2_TiO_3_ at different current densities. The LTOC has the best rate capability. It starts with a specific capacity of 266 mA h g^−1^ at 0.100 A g^−1^ and ends with 200 mA h g^−1^ at 1.000 A g^−1^. High specific capacities of 160, 142, 126, and 118 mA h g^−1^ are obtained at even high current densities 2.000, 3.000, 4.000, and 5.000 A g^−1^, respectively (ESI Section 8†). The specific capacity of LTOC is much higher than that of spinel Li_4_Ti_5_O_12_ (about 160 mA h g^−1^).^30^

The reversible specific capacities of LTOC and coke are 266 and 245 mA h g^−1^ (at 0.100 A g^−1^), respectively (Fig. 5c). The percentage of Li_2_TiO_3_ in LTOC is 35.6 wt% (ESI, Section 2†). From these data, the specific capacity of Li_2_TiO_3_ in LTOC is estimated to be 304 mA h g^−1^, which corresponding to insert/extract 1.25 Li^+^ ions per Li_2_TiO_3_. The results show that Li_2_TiO_3_ could host more Li^+^ ions than Li_4_Ti_5_O_12_.^29^

It is known that Li_2_TiO_3_ is not active as an anode material for lithium ion batteries. The reason is that Li_2_TiO_3_ is an electronic insulator. After supporting Li_2_TiO_3_ nanoparticles over coke, better electron transfer pathways between coke and Li_2_TiO_3_ nanoparticles are established. Since the Li_2_TiO_3_ particles are in nanometer level, the Li-transport distances are shortened. In the redox reaction of Li_2_TiO_3_, the insertion of positively charged Li^+^ could be more easily balanced with the uptake of electrons to compensate Ti^3+^ cations.

## Conclusions

In conclusion, the investigation show that the presence of coke carbon could inhibit the crystal growth of Li_2_TiO_3_ in LTOC composite and the Li_2_TiO_3_ nanocrystals are attached on the surface of coke. The tight attachment of Li_2_TiO_3_ nanocrystals on coke offers an excellent interface for electron exchange between Li_2_TiO_3_ and coke carbon, which might be able to fully activate the Li_2_TiO_3_ nanocrystals as an anode material, which could contribute large specific capacity (304 mA h g^−1^). On the other hand, the introduction of Li_2_TiO_3_ into coke raises the *D*_Li^+^_ of coke, and therefore, improves the rate capability of the material. Hence, the high specific capacity, the low discharge/charge voltage of LTOC *vs.* Li^+^/Li, the high rate capability, and the simple preparation method of LTOC make it a practical anode material to make high rate and high energy density batteries.

## Conflicts of interest

There are no conflicts to declare.

## Supplementary Material

RA-009-C9RA02611H-s001
