# Supplementary material for: An investigation of Li2TiO3–coke composite anode material for Li-ion batteries
Source: RSC Adv. 2019 Jun 5;9(31):17835–40. doi: 10.1039/c9ra02611h (PMC9064618; doi:10.1039/c9ra02611h)
Supplement: RA-009-C9RA02611H-s001 [file RA-009-C9RA02611H-s001.pdf]

Electronic Supplementary Material (ESI) for RSC Advances.
This journal is © The Royal Society of Chemistry 2019

Supplementary Information

An Investigation of Li_2TiO_3-coke Composite Anode Material for Li-ion Batteries

Youlin Liu, Wensheng Li, Xiaoping Zhou*

Section1

The Laser Raman characterization shows that the preheated oil coke has 2 bands the D band at 1360 cm^{-1} and the G band at 1580 cm^{-1}. The ratio of the D band intensity (I_D) to the G band intensity (I_G) is 0.91 (I_D/I_G = 0.91), which indicate that the preheated oil coke is amorphous. The shift of the Raman band at 1580cm−1 to 1562cm−1 (graphite), associated with the degree of graphitization, corresponds to a deviation from a perfect graphite crystal plane (Fig. 1). [*Min Gyu Choia,b, Young-Gi Leea, Seung-Wan Songb, Kwang Man Kim ; Anode properties of titanium oxide nanotube and graphite composites for lithium-ion batteries, Journal of Power Sources 195 (2010) 8289–8296; T.C. Chieu, M.S. Dresselhaus, M. Endo, Phys. Rev. B 26 (1982) 5867 ; M. Endo, C. Kim, T. Karaki, Y. Nishimura, M.J. Mattews, S.D.M. Brown, M.S. Dresselhaus, Carbon 37 (1999) 561*]. The TEM images also show that the preheated coke is not graphitized (Fig. 2 and Fig. 3).

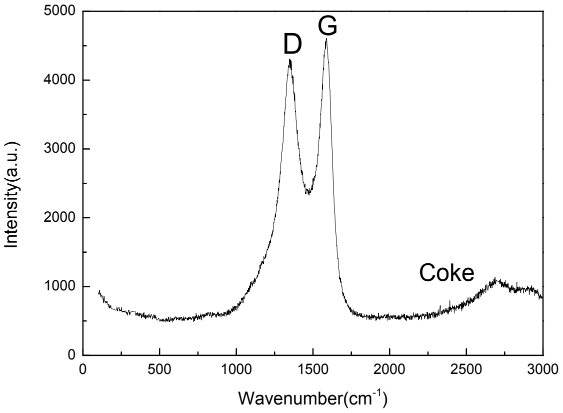

Fig. s1. The Raman spectrum of preheated coke

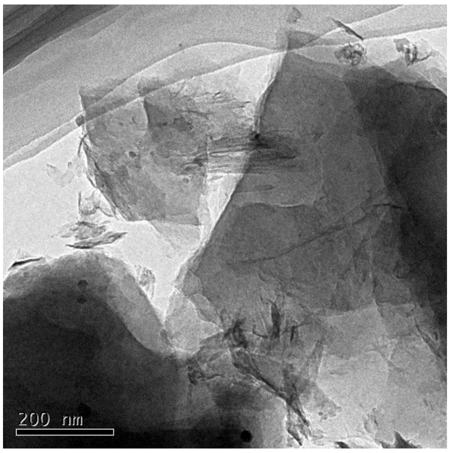

Fig. s2. TEM image of preheated coke

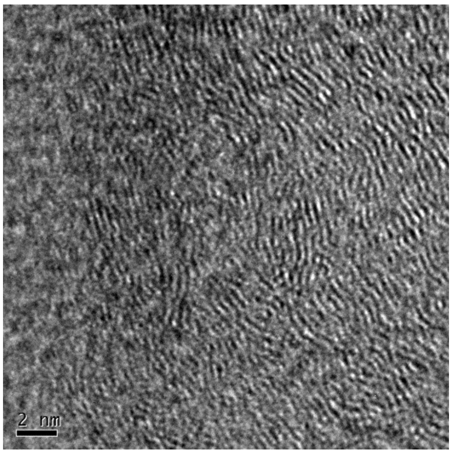

Fig. s3. HRTEM image of preheated coke.

Section 2

The percentage of Li_2TiO_3 in LTOC

LTOC (0.9998 g) was calcined at 800 ℃ in air for 4 h. After calcination, 0.3557 g of white powder Li_2TiO_3 was obtained. The percentage of Li_2TiO_3 in LTOC is 35.6wt%.

Section 3

The EDS analysis indicates that the LTOC contains Al, Si, and S impurities, but not contains N.

Table s1. Impurities in LTOC

Element	***Wt%***	***At%***
CK	55.6	67.92
OK	29.83	27.36
AlK	0.35	0.19
SiK	0.26	0.14
SK	0.81	0.37
TiK	13.15	4.03
VK	0	0
Matrix	Correction	ZAF

Section 4

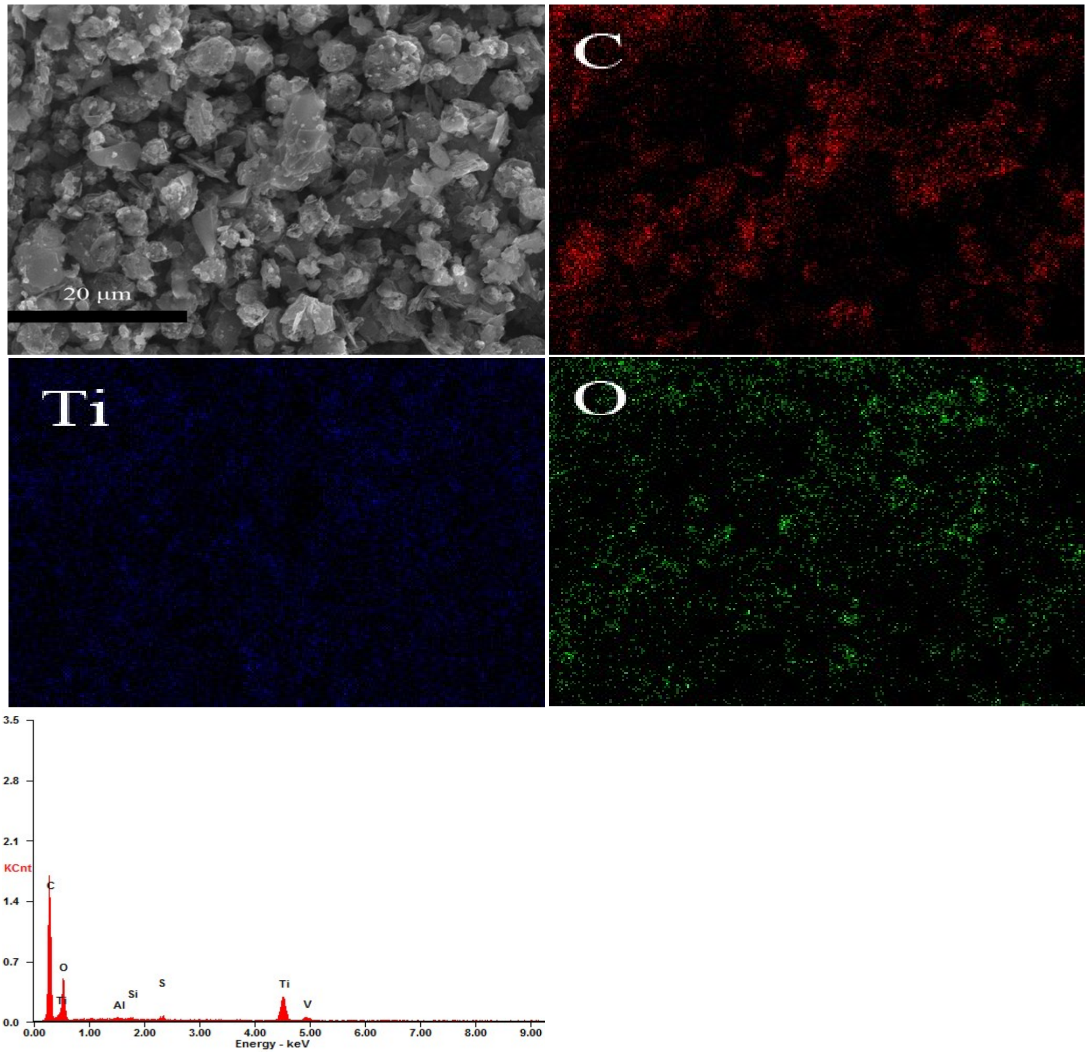

Fig. s4. EDS mapping and the EDS pattern of LTOC

Section 5

(a)

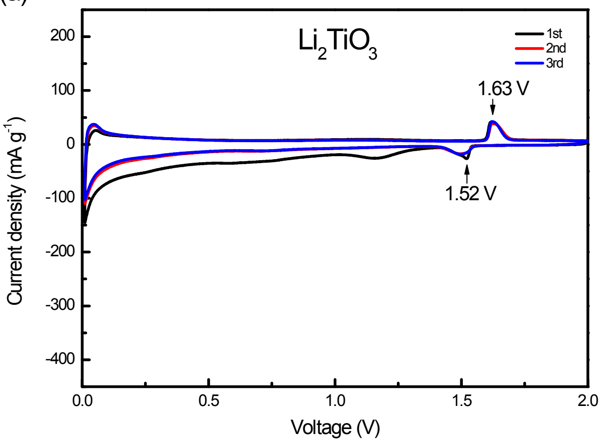

(b)

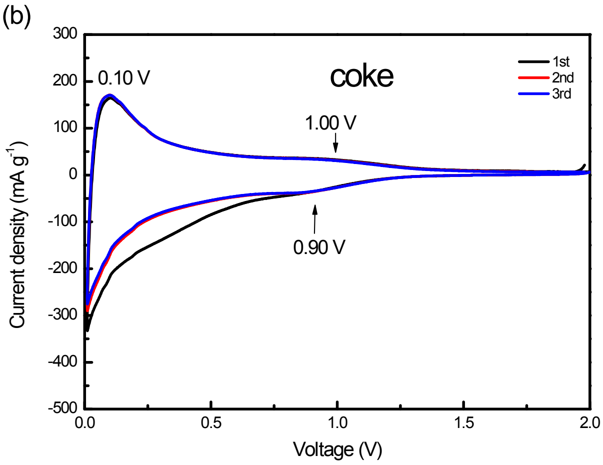

(c)

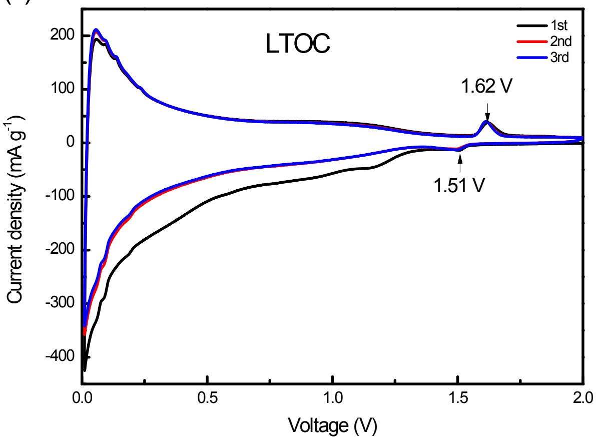

Fig. s5. The CV curves of Li_2TiO_3 (a), coke (b), and LTOC (c) at a scan rate of 0.1 $mV.s^{-1}$, within a voltage window between 0.01 and 2.00 V.

From the CV data of coke, Li_2TiO_3, and LTOC (Fig. S2), the lithium ion diffusion coefficient could be calculated through the Randles-Sevcik equation: $I_p = (2.69\times10^5)\, n^{3/2} A\, D_{Li+}{}^{1/2} C_{Li+} V^{1/2}$ (1)

In equation (1), *A* is the electrode area (cm^2), *n* is the number of electrons transferred, C_{Li+} is the concentration of Li ion (mol cm^{-3}), D_{Li+} is the lithium diffusion coefficient ($cm^2.s^{-1}$), and *V* is the scan rate ($V.s^{-1}$). In our calculation, the area of the electrode is 1.54 cm^2, the concentration of the Li ion is 3.1×10^{-2} $mol.cm^{-3}$, and the value of *n* is 1.

Section 6

Table S2. The impedance data of coke, Li_2TiO_3, and LTOC electrodes

	Re (Ω)	Rs (Ω)	Rct (Ω)
coke	2.29	34.30	59.64
Li_2TiO_3	1.94	27.72	81660

LTOC-3	2.01	28.96	24440

Section 7

From Fig. s6, the cell *$LiNi_{0.6}Co_{0.2}Mn_{0.2}O_2$) cathode –LTOC anode* has the same cathode and anode weights with the cell *$LiNi_{0.6}Co_{0.2}Mn_{0.2}O_2$) cathode-- $Li_4Ti_5O_{12}$ anode*, respectively. Both cells have excess amount of cathode material. However, the cell *$LiNi_{0.6}Co_{0.2}Mn_{0.2}O_2$) cathode –LTOC anode* could store much more energy (the integration of the charge/discharge curves) than that of the cell *$LiNi_{0.6}Co_{0.2}Mn_{0.2}O_2$) cathode-- $Li_4Ti_5O_{12}$ anode.*

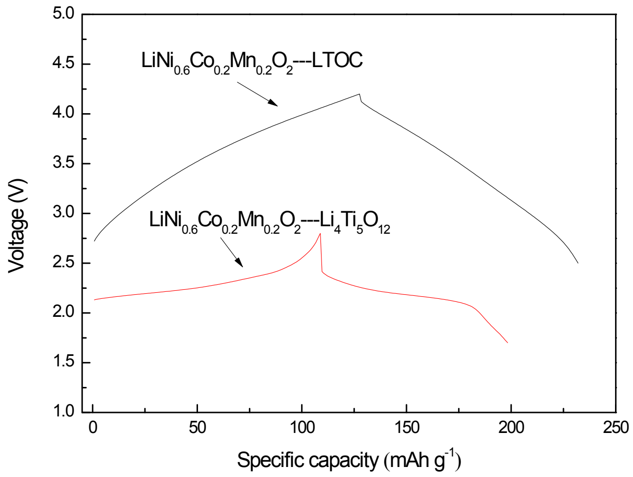

Fig. s6. The full cells: 1) $LiNi_{0.6}Co_{0.2}Mn_{0.2}O_2$) cathode with LTOC anode (charge/discharged between 2.5-4.2 V, at 100mA/g calculated according to the weight of LTOC); 2) $LiNi_{0.6}Co_{0.2}Mn_{0.2}O_2$) cathode with $Li_4Ti_5O_{12}$ anode (charge/discharged between 1.7-2.8 V, at 100mA/g calculated according to the weight of $Li_4Ti_5O_{12}$).

Section 8

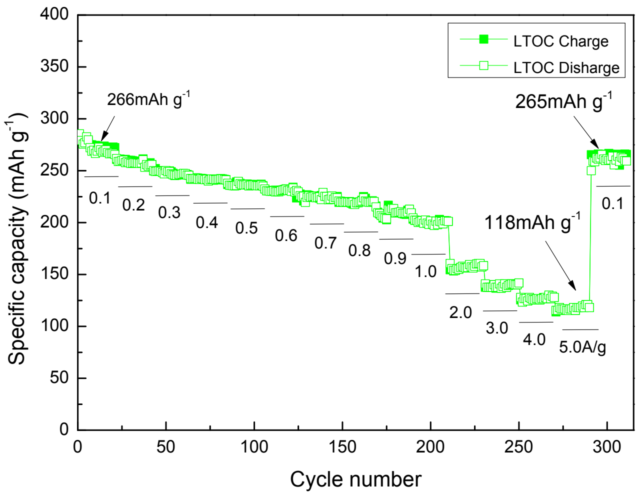

Fig. s7. The discharge/charge rate cycling test of LTOC at different current densities.
